# Acceptability, Usability, and Effectiveness of a Music Video Game for Pain Management: A Crossover Study

**DOI:** 10.3390/healthcare13192439

**Published:** 2025-09-25

**Authors:** Jara Esteban-Sopeña, Javier Bravo-Aparicio, Iria Trillo-Charlín, Alberto Roldán-Ruiz, Hector Beltran-Alacreu, Nuria García-Magro

**Affiliations:** 1 Faculty of Health Science, Universidad Francisco de Vitoria, Pozuelo de Alarcon, 28223 Madrid, Spain; jaramaria.esteban@ufv.es (J.E.-S.); alberto.roldan@ufv.es (A.R.-R.); nuria.garcia@ufv.es (N.G.-M.); 2 Hospital Universitario del Sureste, Arganda del Rey, 28500 Madrid, Spain; 3 Pain, Mental Health, Exercise and Technology Research Group (PAIN+MET), Faculty of Physical Therapy and Nursing, Universidad de Castilla-La Mancha, 45071 Toledo, Spain; 4 Toledo Physiotherapy Research Group (GIFTO), Faculty of Physical Therapy and Nursing, Universidad de Castilla-La Mancha, 45071 Toledo, Spain; iria.trillo@uclm.es; 5 Toledo Physiotherapy Research Group (GIFTO), Instituto de Investigación Sanitaria de Castilla-La Mancha, 45004 Toledo, Spain

**Keywords:** video game, pain, usability, satisfaction

## Abstract

Background: The increasing use of virtual reality (VR) has extended into medical applications, including pain management through immersive mechanisms. This study aimed to evaluate the effectiveness of the *Clone Hero* for reducing pain intensity, threshold and tolerance. Methods: A randomized crossover trial compared three conditions during a cold pressor test in 25 healthy volunteers over 35 years: playing *Clone Hero* (interactive), watching *Clone Hero* (control), or no intervention (placebo). Outcome measures included usability and acceptability (qualitative questionnaire), pain intensity (VAS), pain threshold, pain tolerance, physical activity (IPAQ), and adverse effects. Results: Twenty-five participants completed the study. Overall satisfaction was high, with 92% reporting a positive experience. The *Clone Hero* group showed significantly lower pain intensity scores (4.9 ± 0.49) than the placebo (5.6 ± 0.48; *p* = 0.037) and control groups (6.1 ± 0.42; *p* = 0.004). Pain threshold was higher in the *Clone Hero* group (74.45 ± 20.7 s) compared to the placebo (62.91 ± 18.58; *p* < 0.001) and control (43 ± 14.77; *p* = 0.001). Pain tolerance was also greater (127.6 ± 9.46 s) versus the placebo (*p* = 0.021) and control (*p* = 0.001). No serious adverse effects were reported. Conclusions: Interactive pain management interventions demonstrated high levels of acceptability and user satisfaction, and may enhance pain modulation more effectively than passive or control.

## 1. Introduction

Virtual reality (VR) has rapidly emerged in recent years as an effective strategy for modulating pain perception [1]. By engaging vision, hearing, and proprioception, VR enables patients to redirect their attention, create immersive environments, and develop coping mechanisms that modulate pain processing [2]. Recent studies show that VR increases pain tolerance and reduces pain intensity in clinical procedures and in laboratory models such as the Cold Pressor Test (CPT) [3,4,5].

Distraction is a commonly used technique in clinical settings to increase pain tolerance. Evidence suggests that technology—particularly video games and VR—effectively diverts attention from painful stimuli [6,7], reducing perceived pain compared with non-game or non-VR controls, suggesting that interactivity and cognitive load are relevant elements [8]. The underlying technological mechanism in both strategies involves the creation of a digital environment with which the user can interact whether embedded within the real world through digital elements or experienced as a fully virtual setting [9,10]. This mechanism is based on immersion, defined as the degree of involvement an individual experiences while engaging with a digital game [11]. Immersion can be categorized into three levels: a basic level, where users invest time and effort in playing; an emotional level, where users become affectively involved; and a total immersion level, associated with the sensation of presence [11]. Overall, the extent of user involvement determines how much their attention, awareness, and thoughts shift from the real world to the events occurring within the game.

Immersion depends on five key components: emotional and cognitive engagement, challenge, dissociation from the real world (influenced by factors such as screen size and background music), and the use of game controllers [11,12]. Controllers, in particular, play a crucial role in the degree of immersion, directly influencing the user experience. As such, the user may adopt either a passive observer stance or an active participatory role; the more components involved in the gaming experience, the greater the level of player engagement [12].

To understand how technological immersion modulates pain perception, it is essential to examine the neurophysiological mechanisms involved. According to the neuromatrix theory, the brain processes sensory inputs and uses this information to create a mental representation of the body and environment that guides movement and behavior. The use of applications or video games introduces a surplus of sensory input, which the brain must process, thereby reducing the output of pain signals [13,14].

Additional mechanisms may also be involved, including the activation of the limbic system, which regulates emotions and motivation and is associated with dopamine release [15]. The sympathetic nervous system may be stimulated through the release of adrenaline and noradrenaline, while the parasympathetic nervous system may help reduce the perception of pain by promoting relaxation and rest. The gate control theory proposed by Ronald Melzack and Patrick D. Wall supports this framework by positing that emotional and cognitive states modulate the transmission and perception of pain [16,17].

These neurophysiological mechanisms can be systematically explored through experimental pain models, among which the Cold Pressor Test (CPT) is particularly notable. This procedure involves immersing a limb in ice water to induce pain in a controlled and reproducible manner [18]. Notably, recent studies with large cohorts have demonstrated the efficacy of the CPT in evaluating pain modulation strategies, including those involving VR, which has shown effectiveness in reducing experimental pain contexts [4,19]. VR experiments with CPT consistently show longer tolerance times and lower pain scores during immersion compared with controls [4].

In this clinical trial, the musical tool *Clone Hero* was employed—an interactive application that, to the best of the authors’ knowledge, has not previously been studied in either pain or VR contexts. This tool requires bimanual coordination and sustained attention, demanding a complex motor and cognitive effort from the user. Its game structure allows for the analysis of different levels of distraction and immersion within a musical environment and facilitates the comparison of engagement levels (control, observation, participation). Although no direct evidence has been observed regarding musical video games in an-algesia contexts, related lines of research show benefits of music on pain responses [20,21] and analgesic effects of interactive (non-musical) video games in CPT [8].

In addition to evaluating the tool’s effectiveness in pain modulation, the present study investigates participant usability and satisfaction. The trial was conducted in asymptomatic individuals as part of a proof-of-concept study, offering several methodological advantages. On one hand, it enhances logistical feasibility by simplifying recruitment and ensuring sample homogeneity [22]. On the other hand, it allows technical optimization of the protocol without compromising patient safety or well-being. This approach also yields baseline data that can inform the design of future clinical VR-based interventions, enabling parameter adjustments before clinical implementation. Furthermore, it allows for trials under controlled and homogeneous conditions, minimizing clinical variability. Lastly, it aids in identifying potential adverse effects of the intervention, offering insight for preventing such outcomes in future studies.

The primary objective of this study is to assess the effectiveness of the *Clone Hero* musical application for reducing pain intensity, threshold, and tolerance. Secondary objectives include examining participants’ sociodemographic characteristics, measuring physical activity levels, and monitoring reported adverse effects.

## 2. Materials and Methods

### 2.1. Study Design

A randomized controlled crossover single-blind study with three interventions was conducted between June 2024 and May 2025. The study was approved by the Ethics Committee of Francisco de Vitoria University (13/2024). This study was designed and reported according to CONSORT guidelines [23].

### 2.2. Participants

The study was conducted at two universities in Spain: Universidad Francisco de Vitoria and Universidad de Castilla-La Mancha. A total of 25 participants (13 from University Francisco de Vitoria and 12 from Universidad de Castilla-La Mancha), over 35 years of age were included in the study. All participants were right-handed. Individuals with a history of chronic pain, those experiencing acute pain on the day of the trial or the day before, as well as those who had taken analgesics or opioids within at least 24 h prior to the trial, were excluded. Participants with pre-existing conditions that could affect pain or temperature perception (e.g., Diabetes Mellitus), as well as those with visual or auditory impairments, were also excluded.

Recruitment was carried out through posters placed on university campuses, word-of-mouth among university staff, and snowball sampling, in which enrolled participants recommended other potential volunteers.

Participants who met the eligibility criteria provided informed consent and completed a physical activity test (IPAQ) and a questionnaire to gather sociodemographic data. Then, participants engaged in the intervention in a designated room optimized for comfort and focus. Environmental factors such as temperature (maintained between 20 °C and 25 °C), lighting, and noise levels were regulated to provide a distraction-free. No gift or financial benefit were provided to participants for their involvement in the study.

### 2.3. Interventions

**Figure 1:**
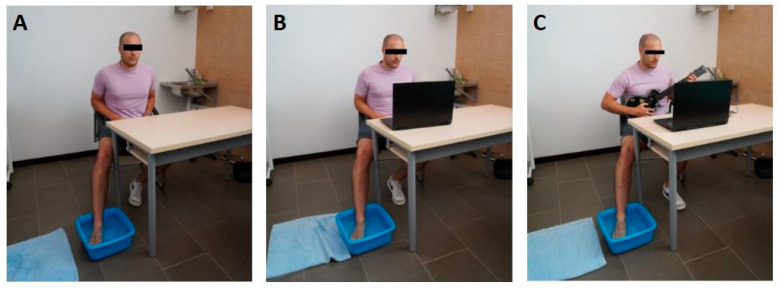
Experimental setup for the three intervention conditions in the crossover design study. (**A**) Control group: foot immersion in cold water without visual or interactive stimuli. (**B**) Placebo group: foot immersion in cold water while watching a prerecorded video of someone playing the *Clone Hero* application. (**C**) *Clone Hero* group: foot immersion in cold water while actively playing the *Clone Hero* musical video game in virtual reality using a guitar controller.

Given that this was a crossover design study with three intervention conditions: an experimental group, a placebo group and a control group (Figure 1). To minimize potential carryover effects between conditions applied on the same day, a washout period of at least 45 min was established between each exposure.

*Clone Hero* group (virtual reality with the musical application “*Clone Hero*”): participants used the “*Clone Hero*” application. During the intervention, participants kept their foot submerged while interacting with the application by playing a 2 min and 35 s song (“I Wanna Be Sedated” by The Ramones) on easy difficulty.

Placebo group: participants watched a prerecorded video of a person playing the same application, without direct interaction with the content.

Control group: participants received no visual or interactive stimulus and only immersed their foot in cold water following the protocol.

#### 2.3.1. Musical Video Game Setup

The musical video game intervention was implemented using the application *Clone Hero* (version 1.0 for Windows) a free rhythm-based music game that allows the incorporation of custom songs and supports various controllers. The equipment setup and configuration were standardized across all sessions to ensure consistency in the intervention experience.

#### 2.3.2. Equipment and Materials

The system included a Windows-based laptop, using its built-in screen and speakers to deliver both visual and auditory stimuli. A USB guitar-shaped controller (Les Pauls Wireless Controller, Redoctane, CA, USA), compatible with *Clone Hero*, was used to simulate instrument play through five colored fret buttons and a strum bar.

##### Hardware and Software Specifications

The laptop used for the interventions had at minimum an Intel Core i5 processor, 8 GB RAM, and an integrated graphics card capable of running the game smoothly (Lenovo ThinkPad L13 Yoga Gen 2, Lenovo, Beijing, China).

*Clone Hero* was configured to run on easy difficulty, allowing for accessible interaction regardless of the participant’s prior musical experience. The selected song for the intervention was “I Wanna Be Sedated” by the Ramones, with a duration of 2 min and 35 s. Audio-visual synchronization was calibrated before each session using the game’s built-in tools.

### 2.4. Pain Induction Protocol

Participants were instructed to immerse their right foot up to the lateral malleolus in cold water maintained between 0 and 1 °C. To maintain this temperature, ice cubes were added to the water, and the water temperature was measured again with a thermometer before each intervention. Before immersion, the ice cubes were removed to avoid direct contact with the subject’s skin.

Pain threshold was defined as the time at which participants verbally indicated discomfort by saying, “It bothers me.” Pain tolerance was recorded as the time at which the participant withdrew the foot due to intolerable pain. Both times were recorded using a digital stopwatch. The person operating the stopwatch remained in the room with the participant but did not interact with them at any point Participants then completed a Visual Analog Scale (VAS) to rate perceived pain intensity. The maximum immersion time was limited to 2 min and 35 s, in accordance with the duration of the selected song used during the intervention and with previous literature, which establishes a maximum limit of 3 min [24].

### 2.5. Outcomes Measures

#### 2.5.1. Primary Outcomes

##### Usability and Acceptability Assessment

A custom qualitative test was also administered to all participants after the intervention designed to assess the usability of the *Clone Hero* video game and their overall satisfaction with the experience. The questionnaire explored aspects such as ease of interaction, engagement, comfort, and whether they found the activity enjoyable or distracting. These measures aimed to provide insight into participants’ subjective experiences and perceptions of each condition.

##### Pain

Pain was evaluated using three indicators: pain intensity, pain threshold, and pain tolerance. Pain intensity was assessed through a Visual Analog Scale (VAS). This scale consists of a 100 mm horizontal line with pain descriptors marked at each end: “no pain” on the left side and “worst imaginable pain” on the right. Patients were instructed to mark the line to indicate the level of pain experienced immediately after the withdrawal from the cold water.

Pain intensity was classified as follows: 0 = no pain, 1–4 = mild pain, 5–7 = moderate pain, and 8–10 = severe pain.

Pain threshold was defined as the moment at which the participant first reported discomfort during the cold-water immersion by verbally stating “It bothers me.” Pain tolerance was defined as the total time the participant was able to maintain foot immersion before voluntarily withdrawing due to intolerable pain. Along with pain threshold, pain tolerance was recorded in seconds using a digital stopwatch, as described in the cold pressor protocol.

#### 2.5.2. Secondary Outcomes

Sociodemographic variables (sex, age, weight, and height) were recorded at baseline, along with physical and psychological variables assessed through the questionnaires described below.

##### Physical Activity

Physical activity was measured as a secondary outcome using the International Physical Activity Questionnaire (IPAQ). This questionnaire consists of seven items evaluating the frequency, duration, and intensity of physical activity performed during the previous seven days, as well as walking and sitting time on a typical workday.

This scale was administered at baseline, together with the initial questionnaires, prior to the first intervention applied. According to the IPAQ scoring protocol, higher scores indicate greater levels of physical activity (high category), whereas lower scores reflect lower levels of physical activity (low category).

##### Adverse Effects Monitoring

Participants were instructed to report any discomfort or adverse effects immediately after each condition.

### 2.6. Sample Size

For the usability study, a target sample size of 15 participants was initially calculated based on prior research in the field of usability and technology-based interventions. This estimate was considered sufficient to explore user experience and detect potential usability and acceptability issues in small-scale studies. However, to account for possible dropouts or incomplete data, a total of 27 participants were ultimately enrolled and assessed [25,26].

### 2.7. Randomization

Randomization was performed using the random number generator “Échaloasuerte” (https://echaloasuerte.com/number; accessed on 27 May 2024). One of the investigators assigned a number from 1 to 3 to each intervention, and the application was then used to randomly generate one of these values. The number obtained determined the corresponding intervention each participant received. In this way, all participants followed different sequences and started with the intervention randomly allocated to them, which minimized potential order effects.

Due to the nature of the interventions, participants were not blinded to their condition. However, outcome assessors were blinded to the order and content of the interventions, ensuring partial blinding at the assessment level.

### 2.8. Procedure

Before the start of the study, all participants received an anthropometric data questionnaire via email, the informed consent form, and the International Physical Activity Questionnaire (IPAQ). All these documents had to be completed and submitted by the participants on the day of the intervention in the research room. Before the start of the intervention phase, participants were allowed to carry out up to three practice attempts with the *Clone Hero* application to familiarize themselves with the gameplay mechanics, regardless of prior experience.

Upon arrival at the research room, a two-minute acclimation period was allowed before the start of the procedure. On each condition, the protocol involved immersing the participant’s right foot in cold water while performing the assigned activity: actively engaging with the musical video game in the experimental condition, passively observing gameplay in the placebo condition, or receiving no stimulation in the control condition. The order of conditions was randomized per participant. During each phase, pain threshold and tolerance were recorded, followed by VAS pain scoring, qualitative feedback and monitoring for adverse effects.

### 2.9. Statistical Analysis

Descriptive statistics, including means and standard error of the mean (SEM), were calculated using an Excel spreadsheet (Microsoft Office Professional Plus 2010, Windows 10). To evaluate differences between variables, appropriate statistical tests were applied depending on data distribution. To evaluate differences among the three interventions, a repeated-measures ANOVA was applied. When significant effects were found, pairwise comparisons were conducted using the Student’s t-test for parametric data and the Wilcoxon test for non-parametric data. The normality of the data was assessed using the D’Agostino–Pearson test. All statistical analyses were conducted with GraphPad Prism software (version 8.0, Windows). Statistical significance was determined by *p*-values and indicated in the figures as follows: * *p*  <  0.05, ** *p*  <  0.01, *** *p*  <  0.001.

## 3. Results

### 3.1. Participant Characteristics

**Figure 2:**
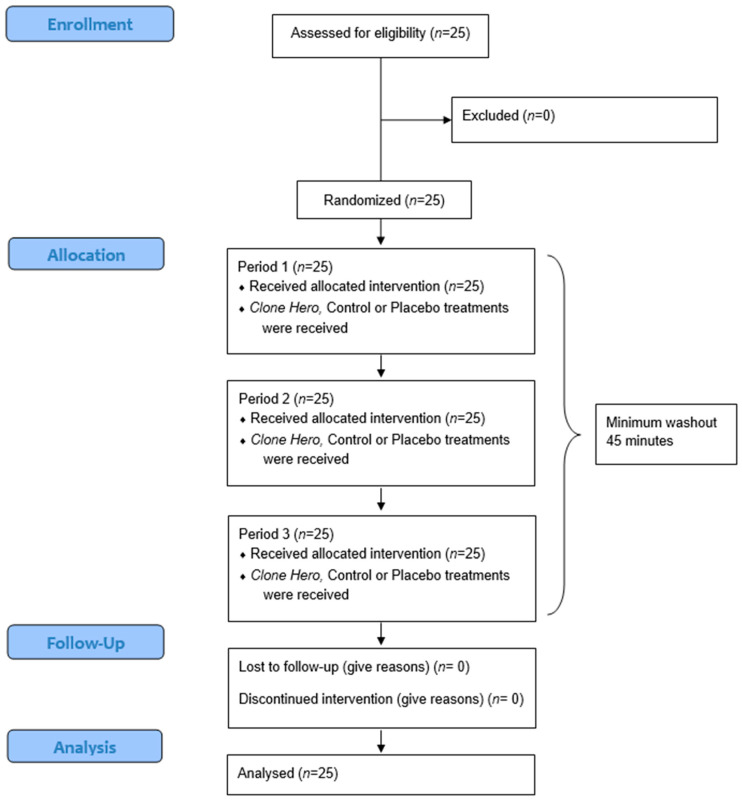
Recruitment flow diagram.

A total of 25 participants with an average age of 45.9 ± 8.4 years, 12 males and 13 females, were qualified and consented after fulfilling the inclusion criteria from June 2024 to May 2025 (Figure 2). All 25 participants completed three interventions and were included in our analysis. Figure 1 shows the recruitment flow diagram of patients through the trial. The baseline characteristics of the patients in each group are presented in Table 1.

### 3.2. Primary Results

#### 3.2.1. Usability and Acceptability Assessment

A total of 25 participants completed a qualitative questionnaire, which included pre-intervention questions regarding prior user experience, and post-intervention questions addressing concentration and distraction as pain modulators, cold tolerance, environmental conditions, prior knowledge of distraction techniques, user perception of innovative methods, and overall satisfaction. All responses are detailed in Table 2.

##### Pre-Intervention Questions

Prior Experience

Most participants indicated no prior experience with the game *Clone Hero* (88%). Reported skill levels for playing the game varied, though “Fair” (40%) and “Good” (16%) were the most common. The majority also stated they had never played string instruments before (80%), although some reported previous familiarity with guitar or similar instruments (20%).

##### Post-Intervention Questions

Concentration and Distraction as Pain Modulators

In total, 68% of participants found it easy to concentrate on the game despite cold exposure. When asked about the influence of concentration on pain perception, 92% of participants agreed or strongly agreed, indicating a positive tendency to recognize the link between attention and perceived pain intensity. Similarly, 92% stated that music and entertainment helped reduce their pain perception. Finally, 80% agreed that distraction reduced pain intensity.

Cold Tolerance

When asked about prior experiences with cold exposure, 88% of participants reported previous contact with this stimulus. Self-reported cold tolerance on a scale from 1 to 10 showed a wide range, with 7 being the most frequently reported value (36%). Regarding whether music and gameplay influenced their response to cold, 96% agreed or strongly agreed, indicating that these elements played a key role in their test experience.

Environmental Conditions

Participants were asked about the impact of the environment during the experimental activity, specifically regarding ambient temperature, furniture, and physical space. 32% reported that environmental conditions affected their experience, while 68% disagreed or were neutral. However, 92% did not suggest any changes to the experimental setup.

Prior Knowledge of Distraction Techniques and User Perception of Innovative Methods

Regarding prior knowledge of distraction as a pain management strategy, 44% of participants reported familiarity, while 56% had no previous knowledge. The overall perception of innovative methods for pain management was positive. In total, 92% agreed or strongly agreed with their effectiveness and potential utility. The remaining 8% disagreed. Most participants (84%) would recommend *Clone Hero* as a future strategy for pain management.

Overall Satisfaction

The level of satisfaction was very high. In total, 92% of participants reported a satisfactory experience. Only 8% expressed a neutral stance, with no participants reporting dissatisfaction.

#### 3.2.2. Pain

##### Pain Intensity (VAS)

**Figure 3:**
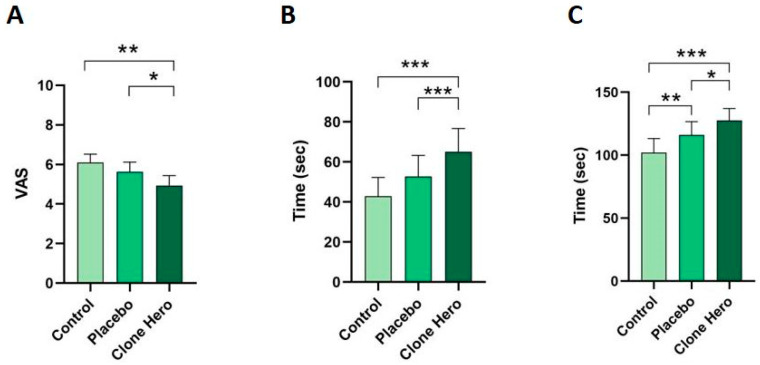
Pain intensity (**A**), pain threshold (**B**), and pain tolerance (**C**) across experimental conditions. Participants in the interactive *Clone Hero* group reported significantly lower pain intensity (VAS scores) (**A**), higher pain threshold (time keeping foot submerged in cold water) (**B**), and greater pain tolerance (total time withstanding the cold stimulus) (**C**) compared to both the control and placebo groups. Statistically significant differences are indicated as follows: *p* < 0.05 (*), *p* < 0.01 (**), *p* < 0.001 (***). Error bars represent standard error of the mean (SEM).

A repeated-measures ANOVA was initially performed, which revealed a significant effect (F(1.845, 44.29) = 5.652, *p* = 0.0078 *). Subsequent statistical analysis using pairwise t-tests identified significant reductions in pain intensity in the experimental condition (Figure 3A). Participants who engaged with the *Clone Hero* (4.9 ± 0.49) reported lower VAS scores compared to both the control group (6.1 ± 0.42) and the placebo group (5.6 ± 0.48). *Clone Hero* participants showed significantly lower VAS scores than those in control group (*p* = 0.0046, **), and those in placebo group (*p* = 0.0367 *). No statistically significant difference was observed between control group and placebo group (*p* = 0.1854).

In addition to statistical significance, effect sizes indicated moderate to large effects (ηp^2^ = 0.191, f = 0.485). Pairwise comparisons also showed meaningful magnitudes of difference (*Clone Hero* vs. control, dz = 0.625; *Clone Hero* vs. placebo, dz = 0.442).

##### Pain Threshold

An initial repeated-measures ANOVA indicated a significant overall effect of condition on pain threshold (F(1.367, 32.80) = 5.486, *p* = 0.0169 *). Pairwise comparisons using t-tests revealed statistically significant differences in pain threshold across the three conditions (Figure 3B). The time participants kept their foot submerged in cold water was significantly longer in the interactive *Clone Hero* group (74.45 ± 20.70 s) compared to both the control group (43.00 ± 14.77 s; *p* = 0.0006, ***) and the placebo group (62.91 ± 18.58 s; *p* = 0.0004, ***). Mean times increased progressively across the three conditions, with the control group showing the lowest pain threshold and the *Clone Hero* group the highest. Non statistically significant differences were found between the control and placebo groups (*p* = 0.02376).

Effect size analysis confirmed moderate to large effects (ηp^2^ = 0.186, f = 0.478), with pairwise comparisons showing strong effects for *Clone Hero* vs. control (dz = 0.790) and *Clone Hero* vs. placebo (dz = 0.822).

##### Pain Tolerance

A repeated-measures ANOVA confirmed a significant effect of condition on pain tolerance (F(1.714, 41.15) = 8.560, *p* = 0.0013 **), with a clear pattern emerged in pain tolerance across groups and statistically significant differences observed in all pairwise comparisons (Figure 3C). Participants exposed to the interactive *Clone Hero* condition withstood the cold stimulus for a significantly longer time (127.6 ± 9.46 s) compared to both the placebo group (116.2 ± 10.46; *p* =0.0210, *) and the control group (102.2 ± 11.12; *p* = 0.0006, ***). Additionally, the placebo group outperformed the control group in pain tolerance (*p* = 0.0087, **).

Effect sizes were again moderate to large (ηp^2^ = 0.263, f = 0.597), with pairwise comparisons indicating meaningful differences (*Clone Hero* vs. control, dz = 0.790; *Clone Hero* vs. placebo, dz = 0.494; placebo vs. control, dz = 0.571)

### 3.3. Secondary Results

#### 3.3.1. Physical Activity (IPAQ)

Sample characteristics include IPAQ scores (Table 3). Based on the IPAQ classification, 40% of participants reported low physical activity, 44% moderate activity, and 16% high activity levels. The total IPAQ scores indicate that the study sample belongs to the moderate physical activity category. Differences were observed between sexes. Among female participants, 46.2% reported moderate physical activity, 30.8% high activity, and 23.1% low activity. In contrast, 50.0% of male participants were classified as moderately active, 33.3% as highly active, and 16.7% as having low activity. Overall, most participants engaged in moderate to high levels of physical activity, with a slightly higher proportion of moderate activity among males.

#### 3.3.2. Adverse Effects

No serious adverse events were reported during or after the interventions. A few participants (*n* = 3) reported mild discomfort related to cold exposure, such as tingling in the immersed foot. These symptoms were resolved spontaneously and did not interfere with the completion of the protocol. No participants withdrew from the study due to adverse effects.

## 4. Discussion

The results obtained from the qualitative assessments suggest that the musical application *Clone Hero* acts as an effective distraction mechanism—an essential factor in the modulation and perception of pain. Participants generally reported that concentrating on the game and engaging with the musical experience helped reduce their perception of pain. The overall satisfaction with the intervention was very high, and most participants expressed a willingness to recommend the tool for future use in pain management. Experimentally, statistically significant improvements were observed in pain intensity, threshold, and tolerance in the intervention group compared to both the control and placebo groups.

These results may be primarily due to the distraction and concentration exhibited by the subject while playing the video game. Musical immersion and the cognitive and motor multitasking demands contribute to modulating the pathophysiological mechanisms of pain by influencing attention [27,28]. The use of a guitar-like controller was particularly well-received, and generated a greater sense of immersion and presence compared to the passive alternative (control group).

Active user participation is a key element in this context: the ability to make decisions and perform concrete actions during gameplay increases the user’s level of concentration and reinforces their sense of participation [29]. In pain situations, divided attention limits the availability of cognitive resources to process nociceptive stimuli. Furthermore, active participation in musical video games like *Clone Hero* can induce a sense of achievement when completing a challenging task. This emotional component stimulates the release of dopamine, a neurotransmitter associated with analgesic effects [29,30]. Successfully completing a song, receiving visual feedback, or finishing a task can generate a rewarding experience that contributes to reducing the subjective component of pain.

In addition, music has a positive emotional impact that can modulate pain perception. Previous studies have demonstrated its effectiveness in reducing pain intensity and associated discomfort [31,32]. These mechanisms can be explained from the perspective of Gate Control Theory, which posits that cognitive-emotional involvement can “close the gate” to pain signals at the spinal level, thereby reducing their transmission to the brain [17,33].

These findings are consistent with previous literature supporting the use of distraction methods and immersive technologies as non-pharmacological analgesic strategies [33,34,35]. The use of virtual reality and similar technologies is on the rise and has proven to be a promising alternative for pain management in both clinical [36] and experimental settings [37]. Evidence also shows that active participation, unlike passive observation, promotes greater cognitive and emotional engagement by the user [34].

Although these findings in healthy participants provide valuable proof-of-concept evidence, caution is required when extrapolating them to clinical populations. Pain in patients is often more complex, influenced by chronicity, comorbidities, and treatment-related factors that may alter both perception and response to distraction-based interventions. Nevertheless, the feasibility and acceptability observed in this study suggest that music-based video games such as *Clone Hero* have potential as adjunctive, non-pharmacological strategies to complement standard care. Future trials including diverse patient groups are needed to evaluate their clinical applicability and effectiveness.

Beyond statistical significance, the magnitude of the effects was moderate to large for both global and pairwise comparisons. This reinforces the clinical and experimental relevance of the findings, suggesting that interactive music-based gaming may provide a meaningful modulation of pain perception. Reporting these effect sizes allows a clearer interpretation of the results in terms of their potential impact.

Moreover, this study addresses a current scientific need: to assess not only the effectiveness of these methods but also their usability and acceptability under experimental conditions [25]. These aspects are essential for ensuring adherence in real-world interventions. These studies also highlight the importance of considering the user’s subjective experience when implementing such technologies [25].

One of the main strengths of this study lies in its methodological rigor and practical applicability. From a methodological standpoint, being a crossover randomized study allowed for intra-subject comparisons, reducing individual bias. Additionally, partial blinding of evaluators helped minimize potential biases in data collection.

The study addresses pain from a multimodal approach, integrating objective measures such as pain threshold and tolerance, and subjective measures such as perceived pain intensity, thus providing a more comprehensive view of the pain experience. The use of standardized protocols and measures is a key element that ensures the feasibility of the design and facilitates its replicability in future research. Finally, the use of the musical application *Clone Hero* represents an innovative alternative. Its accessible, playful, and interactive nature, along with its engaging capabilities, makes it a promising tool for exploring new non-pharmacological methods for pain management.

### Study Limitations and Future Research

This study presents several limitations that should be considered. Firstly, the sample size was small and composed exclusively of healthy participants, limiting the generalizability of the results to real clinical contexts. The lack of full blinding of participants may have influenced their responses. Moreover, the intervention was conducted under controlled laboratory conditions, far from real clinical environments. Other variables, such as participants’ musical preferences, were not considered. Familiarity with the game may also have influenced the level of immersion, the learning curve during the intervention, and the degree of frustration in participants with little or no previous exposure. These limitations open multiple future research avenues that should be explored. Replicating the study with larger and more heterogeneous clinical samples, incorporating different levels of musical difficulty, or allowing song selection to personalize the experience would be advisable.

Finally, although the sample size was sufficient for a usability-oriented proof-of-concept design, we also conducted an a priori power analysis, as recommended by the reviewer. Assuming a medium effect size (η^2^ = 0.06; f = 0.25), α = 0.05, power = 0.80, and correlation among repeated measures = 0.50, the required sample size was *n* = 24 under ideal sphericity (ε = 1.0) and *n* = 28 under a conservative assumption (ε = 0.75). Our sample of 25 participants therefore meets the first scenario and closely approaches the second. While this supports the adequacy of the analyses for medium effects, we acknowledge that the relatively modest sample may still limit power for detecting smaller effects, and the risk of type II error cannot be completely excluded.

## 5. Conclusions

The findings of this study suggest that the musical application *Clone Hero* may serve as a useful distraction strategy for acute pain episodes. The high levels of usability and acceptability reported by participants reinforce its potential as an engaging and well-tolerated non-pharmacological approach. The minimal adverse effects observed support the feasibility of exploring its application in clinical contexts. Further research is needed to determine its effectiveness and scope in real-world healthcare settings, particularly regarding long-term pain management.

## Figures and Tables

**Table 1:** Sociodemographic and baseline characteristics from each group.

Outcome	Value
Age (mean ± SD)	45.9 ± 8.4
Sex	Male 12 (48%)/Female 13 (52%)
Height (mean ± SD)	168.8 ± 9.5
Weight (mean ± SD)	75.2 ± 15.6
Smoker (yes/no)	Yes: 6 (24%)/No: 19 (76%)
Physical Activity (IPAQ)	Low: 5 (20%), Moderate: 12 (48%), High activity: 8 (32%)

**Table 2:** Distribution of responses from the qualitative questionnaire on the usability and acceptability of the *Clone Hero* tool.

**Previous experience**	1. Previous experience with the ***Clone Hero*** game	Yes	12%
		No	88%
	2. Ability to play *Clone Hero*	Good	16%
		Regular	40%
		Bad	44%
	3. Previous experience with guitar or string instrument	Yes	20%
		No	80%
**Concentration and distraction as pain modulators**	4. Ease of concentrating on the game during cold exposure	Totally agree	32%
		Agree	36%
		Neutral	16%
		Disagree	16%
		Totally disagree	0%
	5. Perceived influence of concentration on pain perception	Totally agree	56%
		Agree	36%
		Neutral	4%
		Disagree	4%
		Totally disagree	0%
	6. Impact of music and entertainment on pain perception	Totally agree	60%
		Agree	32%
		Neutral	0%
		Disagree	8%
		Totally disagree	0%
	7. Noticed distraction reduces pain intensity	Totally agree	52%
		Agree	28%
		Neutral	8%
		Disagree	12%
		Totally disagree	0%
**Cold tolerance**	8. Prior experience of pain from cold exposure	Yes	88%
		No	12%
	9. Self-rated cold tolerance (scale 1–10)	0	0%
		1	0%
		2	0%
		3	8%
		4	4%
		5	16%
		6	8%
		7	36%
		8	20%
		9	0%
		10	8%
**Concentration and distraction as pain modulators**	10. Music and gameplay influencing cold response	Totally agree	40%
		Agree	56%
		Neutral	0%
		Disagree	4%
		Totally disagree	0%
**Environmental conditions**	11. Environmental factors affecting pain sensation	Totally agree	12%
		Agree	20%
		Neutral	44%
		Disagree	24%
		Totally disagree	0%
	12. Suggested changes to the experimental setup	Yes	8%
		No	92%
**Previous knowledge of distraction techniques**	13. Awareness of distraction techniques for pain before study	Yes	44%
		No	56%
	14. Perceived innovation of combining music and gaming	Totally agree	40%
		Agree	52%
		Neutral	4%
		Disagree	4%
		Totally disagree	0%
	15. Openness to novel pain relief methods	Totally agree	56%
		Agree	36%
		Neutral	0%
		Disagree	8%
		Totally disagree	0%
	16. Willingness to recommend *Clone Hero* as pain strategy	Totally agree	44%
		Agree	40%
		Neutral	0%
		Disagree	16%
		Totally disagree	0%
**Satisfaction**	17. Overall satisfaction with the experience	Totally satisfied	60%
		Satisfied	32%
		Neutral	8%
		Unsatisfied	0%
		Totally unsatisfied	0%

**Table 3:** Distribution of Physical Activity Levels according to IPAQ classification, overall and by sex.

IPAQ Level	Total Sample (%)	Female (%)	Male (%)
**Low activity**	40%	23.1%	16.7%
**Moderate Activity**	44%	46.2%	50%
**High Activity**	16%	30.8%	33.3%

## Data Availability

The relevant data are available upon request to the corresponding author.

## Abbreviations

The following abbreviations are used in this manuscript:

CTP	Cold Pressor Test
IPAQ	International Physical Activity Questionnaire
VAS	Visual Analog Scale
VR	Virtual Reality
